# Perceptions of deprescribing for patients with limited life expectancy in primary care

**DOI:** 10.1017/S1463423625100686

**Published:** 2025-12-29

**Authors:** Emma C.M. van Aken, Maike S. van der Waal, Saskia C.C.M. Teunissen, Allegonda G. Uyttewaal, Cathelijne Verboeket-Crul, Hanneke Smits-Pelser, Eric C.T. Geijteman, Matthew P. Grant

**Affiliations:** 1 Center of Expertise in Palliative Care, Julius Center for Health Sciences and Primary Care, University Medical Center Utrecht, Department of General Practice, Utrecht, The Netherlands; 2 Academic Hospice Demeter, De Bilt, The Netherlands; 3 Leidsche Rijn Julius Healthcare Center, Parkwijk, Utrecht, The Netherlands; 4 Department of Medical Oncology, Erasmus Medical Center Cancer Institute, Rotterdam, The Netherlands

**Keywords:** deprescribing, end-of-life care, interview study, perceptions, primary care

## Abstract

**Aim:**

The study aims to understand the perceptions of deprescribing in primary care for patients with a limited life expectancy.

**Background:**

In the setting of limited life expectancy, medications may become inappropriate when the possible harms of use outweigh the benefits. Whilst the cessation of potentially inappropriate medications is associated with improved patient outcomes, incorporating this process into routine primary care is poorly enacted.

**Methods:**

Qualitative interview study performed in primary care settings in the Netherlands, including primary care health professionals, patients with limited life expectancy, and their caregivers. Semi-structured interviews were conducted and analysed using inductive thematic analysis.

**Findings:**

Three key themes emerged: (1) facilitating well-being, (2) preventing harm, and (3) dealing with uncertainty. A key goal of mediation use is to facilitate well-being, although the perceptions of this effect may not always match the reality due to changed clinical circumstances. The decision to continue or stop medication is influenced by the wish to prevent harm and to what extent participants find ways to deal with the uncertainties facing them.

Reluctance to deprescribe medications is often related to uncertainties around ceasing medications, lack of clear clinical guidance, and the evolving situation of advanced illness. Integrating these discussions into routine primary care for patients with chronic and incurable illnesses may assist patients and healthcare professionals to address issues around medication use in a proactive manner and promote advance care planning discussions.

## Introduction

Patients in the last year of life use an average of 10 medications, with 80% of these patients using at least one potentially inappropriate medication (Arevalo *et al.*, 2018; Pype *et al.*, 2018; Antonisse *et al.,* 2023). Almost 30% of the patients are prescribed a preventive medication on the day of death (Arevalo *et al.*, 2018). Given the short life expectancy and vulnerability of patients at the end of life, the limited benefits and increased harms of these medications should be carefully considered. Polypharmacy – the use of five or more medications – is associated with increased health-related costs, risk of drug–drug interactions, adverse drug events, unplanned hospital admissions, mortality, and poorer quality of life (McNeil *et al.*, 2016; Masnoon *et al.*, 2017; Muhlack *et al.*, 2017).

Medications may become potentially inappropriate due to the changing clinical circumstances of the patients. The medication’s time to benefit may exceed the predicted life expectancy, the initial indication for which it was prescribed may no longer be present, or the treatment goals of the patient may have changed. Thus, there may be limited or no potential benefit to the patient from ongoing use. Examples of potentially inappropriate medications in patients with limited life expectancy are cholesterol-lowering, antihypertensive, proton pump inhibitors, and antidiabetic medicines (Lindsay *et al.*, 2015; Antonisse *et al.,* 2023; Chae *et al.,* 2022).

A potential way to limit the use and risks of potentially inappropriate medication is to engage in deprescribing. Deprescribing is the act of reducing or stopping medication when they are deemed to be no longer appropriate to the patient’s situation or needs, with the aim of improving the patient’s quality of life (McNeil *et al.*, 2016). Deprescribing can have beneficial effects, for instance, a better overall medication adherence, lower potential adverse events, and a reduction of financial costs (Reeve and Wiese, 2014; Shrestha *et al.*, 2020). Despite the advantages associated with deprescribing, incorporating this process into routine primary care is challenging, as it is time-consuming, may be challenging to discuss, and is often lower priority than other medical issues (Scott *et al.*, 2015; Palagyi *et al.*, 2016). Geijteman *et al.* (2018) found that 73% of physicians perceived that patients with limited life expectancy used too many medications. A questionnaire study detailed that almost all patients with chronic morbidities were willing to discontinue medication (Geijteman *et al.*, 2016).

The attitudes and beliefs of patients and health care professionals towards deprescribing influence their willingness to engage in these discussions and subsequent treatment decisions (Geijteman *et al.*, 2016; Lundby *et al.*, 2019). This study aims to understand the perceptions of health care professionals, patients, and caregivers about deprescribing in primary care for patients with a limited life expectancy. Through addressing this topic, the study plans to fill the gap in literature concerning these perspectives of deprescribing in palliative care populations, in order to understand how these attitudes may be addressed in clinical practice in primary care.

## Methods

### Study design

This qualitative study was performed using semi-structured interviews with patients, carers, and primary care health professionals informed by thematic analysis. Reporting was conducted according to the Consolidated Criteria for reporting qualitative research checklist (Tong *et al.*, 2007). The study was reviewed by the institutional review board of the UMC Utrecht (21/498), who did not consider this research subject to the Medical Research Involving Human Subjects Act of the Netherlands.

### Setting and recruitment

This study was conducted in primary care health settings in Utrecht, The Netherlands. Study participants were patients with a limited life expectancy, their caregivers, and primary care health professionals, with data collected between September 2022 and April 2023. General eligibility criteria included age over 18 years, ability to speak Dutch and provide consent. Inclusion criteria for patients consisted of having cancer or advanced chronic illness with a limited life expectancy and experience of polypharmacy. Healthcare professionals (HCPs) identified patients with a limited life expectancy through use of the surprise question (van Lummel *et al.*, 2022). Patients or caregivers with cognitive impairment were excluded.

Participants were recruited by convenience sampling and invited to participate in the study. They were provided with an overview of the research, an invitation to participate and a copy of the information sheet. Written consent was obtained for all patients and caregivers. HCPs were also able to provide verbal consent for telephone or video interviews. Interviews were conducted by a post-doctoral researcher (MvdW) and a junior researcher (EvA), who received formal supervision and training in the conduct of qualitative interviews and analysis by a post-doctoral researcher (MG) specialised in qualitative methods.

### Data collection

Demographic and clinical (for patients with cancer) characteristics were collected, including gender, age (patients), treatment intent (patients), and clinical role (staff). All interviews were semi-structured in nature and were adaptive to the conversation and experiences of the participants, following an interview guide (appendix 1). This guide was developed by the study team which consisted of experienced qualitative researchers, general practitioners (GPs), and nurse specialists. The guide consists of four major lines of enquiry: needs and limitations, practical process, identification of patients and timing, and roles and responsibilities. Data saturation was defined as the point at which no new themes emerged, as described by Saunders *et al*. (2018). Data saturation was predetermined in accord with the four major lines of inquiry, and reached when all four lines were saturated. Given the small number of patients included in this study, an additional patient interview was conducted after data saturation was reached, further confirming the non-emergence of new themes. The interviews were audiotaped and transcribed verbatim and were supplemented by the researcher’s field-notes.

### Data analysis

Data were analysed by three members of the research team (EvA, MG, and MvdW), conducted as an iterative process alongside data collection. Thematic content analysis as described by Kiger and Varpio was employed for data analysis (Kiger and Varpio, 2020). The first step in the analysis process involved familiarisation with the data through transcribing and listening to the interviews. The researchers then generated initial codes from this data. Researchers independently analysed the transcripts and constructed thematic categories from the initial codes of the data. Disagreements in the emerging themes were resolved by group discussions. The themes were then subsequently restructured by EvA, followed by additional group discussions to refine the themes until full consensus was reached among the research team (EvA, MG, MW, ST, CVC, GU, HSP, and EG).

## Results

A total of eighteen interviews were completed, including six patients and caregivers, and twelve HCPs in primary care. The group of HCPs consisted of GPs, nurses, and pharmacists.

**Figure 1.**
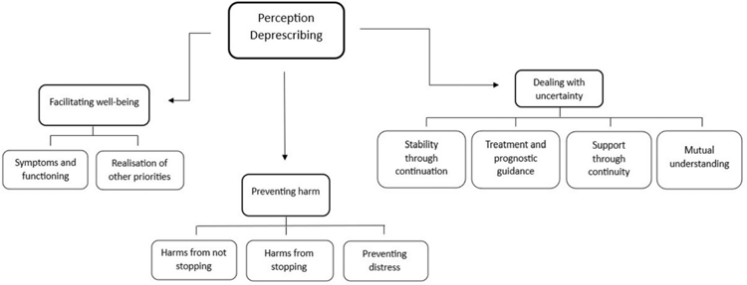
Thematic tree.

Three key themes emerged that detailed the perceptions of deprescribing medication in patient with limited life expectancy in primary care: (1) facilitating well-being, (2) preventing harm, and (3) dealing with uncertainty. The organisation of these themes and subthemes is presented in Figure 1.

### Facilitating well-being

Physical and mental well-being were described as key goals of medication use. HCPs aimed to provide care consistent with patient’s needs to promote quality of life. Corresponding quotes are presented in Table 1.

**Table 1.** Quotes facilitating well-being

*Symptoms and functioning* *I use it to reduce and prevent symptoms. Especially the pill for cancer works, I don’t have pain anymore. – Patient 3* *‘I [patient] turned 94 years old. It’s been good, why am I still taking 15 pills a day? Is this necessary?’ Then you have interesting conversations. ‘Indeed, what are we actually doing? Half of the tablets you take are there to prolong your life and I hear you saying very clear, that you do not really want that anymore.’ – Pharmacist 3* *Realisation of other priorities* *Medication is not as important as mental well-being. Human contact is far more important. – Patient 2* *I think you should not just do a medication review, but do it more broadly. For example, also involve a dietician to keep the nutritional status right. – Pharmacist 2*

#### Symptoms and functioning

Participants described the positive effects of medication through treating health conditions and symptoms, translating to better functioning and quality of life. For patients, medication alleviated symptoms such as pain and were vital to ensure well-being. Medications that did not improve well-being were perceived as ‘unnecessary’ and considered candidates for deprescribing. Related discussions tended to centre around the question whether medication usage was beneficial to pursue in the context of the patient’s limited life expectancy. Patients preferred to take as little medication as possible when they added minimal value to medication use in the last phase of life. Others strongly adhered to their medication usage, perceiving that they stabilize their health situation or extended the last phase of life.

#### Realisation of other priorities

Participants described other priorities that promoted overall well-being. For example, social relationships were mentioned as something that took precedence over the use of medication. Participants perceived that other interventions, such as lifestyle changes, may offer far greater benefits to realise these priorities.

### Preventing harm

Participants wished to prevent harm, which could be both a reason to continue or stop medication. These attitudes were largely dependent on the participant’s experiences with medication, including adverse events and effectiveness. Corresponding quotes are presented in Table 2.

**Table 2.** Quotes preventing harm

*Harms from not stopping* *You stop medication if someone experiences a lot of side effects and the medication has limited added value. But you continue when there is a limited added value, but no complaints. That is rational. – Pharmacist 2* *Harms from stopping* *What hinders me in deprescription, is that I once had a woman with terminal lung cancer staying in a hospice. Her anticoagulants were stopped, upon which she developed a thunderous CVA resulting in hemiparesis and loss of ability to speak. I understand the idea that you can stop blood thinners, cholesterol reducers and ani-hypertensives. On the other hand, it really sucks when you just hit the case where complications occur and then I think with the blood thinner I can relate it to that directly. – General practitioner 3* *Preventing distress* *Yes, it really is a sensitive topic, because you are implicitly reflecting that the patient has nothing more to gain from medication and that you are indicating that you expect the patient to not live much longer. – General Practitioner 3* *I can also imagine that there are elderly people who say ‘if they start taking pills off, I’m written off’. In fact, I know people who think like that, especially among the elderly. They then think ‘oh, they are definitely not going to treat me anymore. Then I will probably have to die.’ That’s a fear people do have, especially in this society. That people feel written off. –Practice nurse 1 + 2*

#### Harms from not stopping

Participants detailed several reasons to deprescribe medication with the aim of preventing harm. These included having to use large quantities of medications, loss of ability to swallow, and the presence of side effects. They identified that these issues outweighed the advantages of continued medication use. For others, the use of many medications had become ingrained and normalised through long-term use, in which case they often did not identify the potential adverse effects as problematic.

#### Harms from stopping

When deprescribing did occur, poor or adverse outcomes that occurred in the timeframe were deemed to be causally linked to the stopping of medication. Because of this, many HCPs perceived it safer to continue medications than to actively deprescribe. Lack of clear guidance about the possible advantages and disadvantages about (dis)continuation of medication contributed to this perception. HCPs described being most wary of deprescribing anticoagulants.

Patients indicated similar fears of harm when deprescribing medication, often related to the loss of the stability of their situation. They were worried that stopping medication may result in the return of symptoms, bad outcomes, or could hasten the end of life.

#### Preventing distress

HCPs described having difficulties in discussing deprescription, due to the perception that these conversations are confronting and implicitly communicate that the end of life is approaching. They understood that these conversations could diminish feelings of hope or control in patients who insist on continuing medication. HCPs described that initiating deprescribing discussions might signify that HCPs have ‘given up on them’.

### Dealing with uncertainty

Participants detailed uncertainty related to the deprescribing process and the evolving situation of advanced illness and sought ways to deal with these uncertainties. Corresponding quotes are presented in Table 3.

**Table 3.** Quotes dealing with uncertainty

*Stability through continuation* *Another group really adheres to medication, because it provides stability and safety. ‘Because the cardiologist told in 1983 that I could never live without this drug.’ – Pharmacist 2* *He had the idea that all the medication was necessary. ‘I have been taking it for so long, maybe it will make me last longer…’ She [nurse specialist} indicated that: ‘From what you are taking, you can leave out a lot of things. It has no use.’ ‘I just go on with it.’ he said. I think that is still a point of him thinking he was postponing death. He didn’t want to stop. – Caregiver 1* *Treatment and prognostic guidance* *Still the uncertainty of prognosis. For example, a woman with COPD, it would not surprise me if she is no longer there in one year’s time. But at the same time, neither if she would still be there in two years. That does not really help me any further. Thus, I am not going to say now, I am going to stop all kinds of medicines for her. I am not sure that her prognosis will be shorter than a year. That she really will not benefit from medication anymore. – General practitioner 3* *Support through continuity* *I notice in our organisation that the doctors take their role very serious here. The fact that we [practice nurses] are sometimes running behind and not being involved is a pity. I suddenly read in the file that something has changed. We see that and think: “Oh hey, that has been done”. So we are constantly one step behind. I find that very unfortunate, but it does happen sometimes.— Practice nurse 1 + 2* *All the steps have to be clear, who does what. So that if you discuss it with the patient and someone indicates that they want it [deprescribe medication], that you will not feel like you do not know how you will control it, actually doing it so to speak. Because it is a sensitive topic, continuity of care is needed. – Practice nurse 3* *Mutual understanding* *I am focused on building a relationship with the patient. If I am going to change medication, they have to trust me. So I am always trying to understand the patient’s view on their medication. What do they think of it? Which concerns do they have? I am asking how it is for the patient. I think that in this way, patients will more easily talk about it and are less afraid. – Pharmacist 3* *Well for me, the approach is the explanation, just giving it words. But that’s not always easy with simple-mined people. See, where you have to use a lot of practical language, it is a bit more difficult. Or people who think it’s easy, and say I’ll do what you say. You still want people to understand you. That it is not a matter of giving you [the patient] up. No, it has more disadvantages than advantages. – Nurse specialist 1*

#### Stability through continuation

Medication continuation was perceived by participants as a manner to deal with the uncertainties caused by their uncertain clinical trajectory. Medication use could be perceived to represent stability or as something that may prolong life. Patients might insist on the continuation of medication in denial of their poor prognosis. HCPs described that it could confuse patients if they are asked to stop medication that they were previously told was vital for their treatment.

#### Treatment and prognostic guidance

The deprescribing of medication occurs within the milieu of treatment and prognostic uncertainties. HCPs wished for evidence to justify and inform their decisions, but current protocols and guidelines were described as lacking the required information about the disadvantages of (dis)continuation of medication in the context of a limited life expectancy. This uncertainty was compounded by the limitations in estimating life expectancy. This lack of clinical guidance translated to reluctance of HCPs to start the deprescribing conversation, fearing the possibility to say or do something incorrect which may cause harm.

#### Support through continuity

Participants detailed numerous uncertainties arising through medications being managed through interdisciplinary collaboration. HCPs mentioned that they did not always feel involved in the prescribing process and therefore lack overview of the situation.

Participants expressed a wish for clarity about the process and the different roles and responsibilities for HCPs. Participants identified the GP as being ideally responsible for initiating deprescribing due to the continuous care relationship and overview of the patient’s situation. However, participants described that many HCP may fulfil this role, especially when they are trusted by the patient.

#### Mutual understanding

Patients detailed the need to feel heard and understood in discussions, which enabled trust and a better appreciation of the treatment goals through which deprescribing could be tailored. HCPs wanted to understand the patient’s needs and wishes, and then could decide together whether or not to discontinue medication. Patients facilitated understanding by taking an active role in these discussions.

When patients were engaged in these discussions about their changing situation, HCPs described confidence in addressing deprescription. In situations where patients might not be empowered or capable to fully engage in treatment discussions, participants perceived that deprescribing was hampered by a lack of mutual understanding. Cultural difference between HCPs and patients were also described as a barrier to communicating each other’s needs and wishes.

## Discussion

This qualitative study describes the perceptions of HCPs, patients, and caregivers in primary care about deprescribing for patients with a limited life expectancy. These discussions were often related (both implicitly and explicitly) to the life goals of the patient, including their physical well-being and social connections. Participants identified that when medications did not meet these goals or improve the patient’s quality of life, ceasing their use should be considered. Focusing on these goals when discussion medication use could be used as a fulcrum to facilitate deprescribing.

Despite deprescribing being identified as having many benefits, patients, caregivers, as well as HCPs were reluctant to initiate these discussions. This was often related to perceived uncertainties regarding the (dis)advantages of deprescription, the deprescription process, and the evolving situation of advanced illness. Participants expressed these uncertainties by finding different ways to deal with these, described in different subthemes, that were often a form of gaining control. The deprescribing discussions were avoided when this form of control and thus certainty could not be achieved.

### Implications for practice

One of the main insights from this study is that participants tend to associate deprescribing with impeding death. HCPs were reluctant to initiate the deprescribing discussion due to the fear to cause distress by confronting patients with conversations involving future care planning and the end of life. Discussing the cessation of medication was frequently perceived as implicitly communicating that prognosis might be short, while HCPs were often uncertain about the life expectancy. HCPs perceived that addressing this issue may signify to patients that they are ‘giving up on them’. This reflects the results of (Dees *et al.*, 2018) where deprescribing medication could feel like diminishing a patients feeling of hope, while maintaining hope was seen as something essential. Hancock *et al.,* 2007 and Bernacki and Block (2014) described that HCPs were reluctant to engage in these discussions for fear of causing emotional distress to the patient, as these conversations frequently addressed end of life issues. However, the perception of patients and loved ones through multiple studies did not confirm this, and existing evidence describes that early end-of life discussions are not necessarily equated with anxiety, hopelessness, and may even reduce these concerns (Detering *et al.*, 2010; Bernacki and Block, 2014; Tjia *et al.*, 2017). Early future planning discussions, including deprescribing medications, is necessary to involve patients in thinking about their care-goals and to act on these goals.

How deprescribing is communicated is key to its implementation, and can address the perceptions of deprescribing, and reduce the uncertainties for patients, caregivers and HCPs. For example, the word ‘deprescribing’ may be perceived as something negative, which may raise misconceptions about the intention of the process. ‘Goal Concordance Prescribing’ has been developed as a positive messaging approach which tries to avoid the word deprescribing, because in patients with limited life expectancy the goal is not only to deprescribe medication, but to achieve the goal of improved quality of life (Tjia *et al.*, 2021). Discussions about how to tailor medication should focus on what aspects of quality of life are important for the patient, rather than the message that taking medication is no longer useful given the short life expectancy. Ideally these discussions should occur early in palliative trajectory, to prepare patients for medication deprescribing and to normalize the routine discussions around the patient’s goals of care.

The shared decision making approach implemented by (Jansen *et al*., 2016) might be highly applicable in deprescribing discussions to take into account personal preferences and promote engagement in the decision-making process (Kuosmanen *et al.*, 2021; Tjia *et al.*, 2021). Participants emphasised the importance of a bond of trust and understanding between HCPs and patients in order to initiate deprescribing. These findings are supported by (Murray and McCrone, 2015; N J Ailabouni *et al.*, 2016; Alwidyan *et al.*, 2023) who found that a preferred approach takes into account the personal perceptions and preferences of the patient, through which trust between HCPs and patients can be enhanced. Trust between patients and primary care professionals is facilitated by having adequate time, open discussions of patient concerns, understanding patient preferences, and having a longitudinal care relationship (Murphy and Salisbury, 2020). Whilst this trust that is often built over many consultations, (Murphy and Salisbury, 2020) suggested that it may be developed even in single consultations through attentive listening, displaying empathy, and clinical competence. Understanding the breadth of the patient’s concerns and preferences for care is vital to deprescribing discussions, as once these are understood, can medication use be optimally discussed to focus on what is most appropriate to meet the patient’s goals and is most likely to improve their quality of life. Although most patients wish to be involved in the decision making process, their participation depends on their knowledge and acceptance of the disease process (Belcher *et al.*, 2006; Bélanger *et al.*, 2011). The willingness and capacity to participate in these decisions might fluctuate over the evolving illness trajectory (Kuosmanen *et al.*, 2021). As the patient’s situation changes, it may be useful to assess patient’s willingness to participate in the deprescribing discussion. Future research could examine how to better involve patients in the decision-making process of deprescribing and may inform the development of communication training for HCPs, including word choice and framing.

These data describe the numerous uncertainties surrounding the deprescribing process, foremost of which was a lack of guidance in prognostication and which medications to deprescribe and when. HCPs noted that current clinical guidelines lack required information about the (dis)advantages of medication (dis)continuation in the context of a limited life expectancy, making them reluctant to deprescribe certain medication (Schuling *et al.*, 2012; Alwidyan *et al.,* 2023). In the past decade a number of guidelines and tools have been developed to provide clinical guidance in this context, including ‘OncoPal’ for patients with cancer and ‘STOPPFrail’ for frail elderly patients with life limiting illness (Lindsay *et al.*, 2015; Lavan *et al*., 2017). However, such guidance is quite general, and do not quantify the risk verse benefit ratio of these medications or provide advice for specific situations of the patient (Reeve *et al.*, 2018).

### Strengths and weaknesses

Data analysis for this study was conducted as a dialectic process involving three researchers, through which the inductive coding and resultant thematic structure was developed. This process incorporated the researchers foregrounding their pre-existing understandings and interpretations and critically reflecting upon their influencing on the resultant analysis. Including patients, caregivers and HCPs from a wide array of roles in primary care was a strength of this study, enabling deprescription to be understood through many perspectives. Whilst the majority of participants were HCPs, sufficient patients and carers were included to reach data saturation. We utilised a convenience sampling approach to maximise recruitment, and utilised a wide network of participants to ensure that there was diversity of participant roles, work settings, and experience to maximise the generalisability of the findings. Verbal informed consent was deemed appropriate for interviews with health professionals, as the risks of participation were negligible and we aimed to limit burdens for participation. The results resonate with previous literature, and reflect the unique influences present in the primary care context. The results of this study reflect the socio-cultural and organisational setting of Dutch primary care, and cannot be generalised to other countries or contexts.

## Conclusion

This study describes the perceptions of HCPs, patients and caregivers about deprescribing in primary care for patients with limited life expectancy. Reluctance to deprescribe medications is primarily related to the many uncertainties regarding the effect of deprescription, confounded by the evolving situation of patients. The decision to cease medications is influenced by the wish to prevent harms associated with (dis)continuing medication. The use of guidelines, continuity in interdisciplinary collaboration and mutual understanding between HCPs and patients are ways to ameliorate these uncertainties and facilitate deprescription, although enacting these changes is challenging in daily practice. The deprescribing discussion should not only aim to deprescribe medication but requires a broader approach that incorporates the patient’s preferences, functioning and other priorities in order to achieve the goal of improved quality of life. Through engaging early in these conversations with patients may enable medications to be reviewed and adapted appropriately throughout the patient’s illness trajectory.

## Supporting information

van Aken et al. supplementary material van Aken et al. supplementary material

## Supplementary material

The supplementary material for this article can be found at https://doi.org/10.1017/S1463423625100686
