# Supplementary material for: Perceptions of deprescribing for patients with limited life expectancy in primary care
Source: Prim Health Care Res Dev. 2025 Dec 29;27:e2. doi: 10.1017/S1463423625100686 (PMC12780418; doi:10.1017/S1463423625100686)
Supplement: van Aken et al. supplementary material [file S1463423625100686sup001.pdf]

APPENDIX 1 INTERVIEW GUIDE

Health care professionals (HCPs)

I	Needs and limitations	In case of a patient with limited lifetime expectancy (less than one year) due to terminal illness or very advanced age:<br>1. To what extend can you imagine a consultation with you or a colleague to discuss cessation of certain medicines?<br>2. What could be reasons to do a medication review?<br>3. What factors limit you or your colleague to do such a medication review?<br>4. What helps you or your colleagues to have such a conversation?<br>5. What do you consider easy or difficult when discussing tapering medication use in this context?
II	Practical process	1. How could medication review and subsequent deprescription fit in the daily care processes?<br>2. What steps would you take when considering deprescribing?<br>3. What medication would you consider stopping and why?<br>4. How do you decide what medication to select for deprescribing?
III	Identification of patients and timing	1. Who are eligible for medication review?<br>2. When is deprescription appropriate? Does timing differ for specific medicines?<br>3. How do you or would you integrate the context of limited life expectancy?<br>4. How can you select patients for medication review?
IV	Roles and responsibilities	1. To what extend are you equipped with enough knowledge and skills to perform deprescribing?<br>2. Who do you consider most appropriate to perform the process of deprescribing?

		3. Could you indicate the desirable role division for this process?<br>4. Do you envision a role for practice nurses, pharmacists, or other care providers?

Patients and caregivers

I	Needs and limitations	Imagine, yourself or a loved one to have limited lifetime expectancy (less than one year) due to terminal illness or very advanced age:<br>1. Would you consider when your medication is then reviewed by an HCP expert?<br>2. To what extend can you imagine a consultation with an HCP to discuss cessation of certain medicines, for example when the risk of harm outweigh the benefits?<br>3. What factors limit you to have such a conversation<br>4. What helps you to have such a conversation?<br>5. What do you consider easy or difficult when discussing tapering medication use in this context?
II	Practical process	6. How would you like consultations about medication use to take place?<br>7. Where would you want these conversations about medication use to take place (at home, at the general practice, …)?
III	Identification of patients and timing	8. When do you consider it to be appropriate to discuss medication use?<br>9. To what extend do you have an idea which medicines could be appropriate to stop and why?
IV	Roles and responsibilities	10. Who would you prefer to discuss medication use with? Consider for example your GP, practice nurse, pharmacist, medical specialist, …<br>11. Who would you not like to discuss medication use with?
